# Stable Carbon Isotope Evidence for Neolithic and Bronze Age Crop Water Management in the Eastern Mediterranean and Southwest Asia

**DOI:** 10.1371/journal.pone.0127085

**Published:** 2015-06-10

**Authors:** Michael P. Wallace, Glynis Jones, Michael Charles, Rebecca Fraser, Tim H. E. Heaton, Amy Bogaard

**Affiliations:** 1 Department of Archaeology, University of Sheffield, Northgate House, West Street, Sheffield, S1 4ET, United Kingdom; 2 Institute of Archaeology, University of Oxford, 36 Beaumont Street, Oxford, OX1 2PG, United Kingdom; 3 NERC Isotope Geosciences Laboratory, British Geological Survey, Keyworth, Nottingham, NG12 5GG, United Kingdom; University of Florence, ITALY

## Abstract

In a large study on early crop water management, stable carbon isotope discrimination was determined for 275 charred grain samples from nine archaeological sites, dating primarily to the Neolithic and Bronze Age, from the Eastern Mediterranean and Western Asia. This has revealed that wheat (*Triticum *spp.) was regularly grown in wetter conditions than barley (*Hordeum *sp.), indicating systematic preferential treatment of wheat that may reflect a cultural preference for wheat over barley. Isotopic analysis of pulse crops (*Lens culinaris*, *Pisum sativum *and *Vicia ervilia*) indicates cultivation in highly varied water conditions at some sites, possibly as a result of opportunistic watering practices. The results have also provided evidence for local land-use and changing agricultural practices.

## Introduction

In the dry regions where early agriculture and early civilisations first developed, water management practices, especially irrigation, were central to agricultural innovation and the rise of complex urban societies [1–5]. The reliable identification of past water management is therefore crucial to our understanding of these economic and socio-political changes. These management practices can operate at a variety of scales, however, ranging from massive irrigation systems extending over hundreds of kilometres to small-scale interventions by individual farmers. Even within the same system, the level of watering may vary from crop to crop, farmer to farmer or even field to field.

Crop water status can be inferred from stable carbon isotope analysis of charred archaeobotanical remains. The technique is based on the principle that discrimination against the heavier carbon isotope ^13^C is greatest when stomata are open [6,7], which in turn is closely linked to the availability of water during carbon fixation [8,9]. Various researchers with an archaeological focus have sought to refine the relationship between water availability and stable carbon isotope ratios for crop species in the Mediterranean region and southwest Asia [10–15]. Based on such studies a relationship between stable carbon isotope ratios and water availability has been established, as well as the limitations of this as a method for inferring past crop water status. This relationship has been used to infer crop water status from ancient charred grain [16–24].

In dry regions, scarcity of water is usually the main limiting factor on plant growth and, therefore, arable production. In these regions, the water status of a crop relates not only to climate but also to the agricultural strategies employed to minimise the harm caused by aridity. Indeed, crop water management is likely to have been at least as important for improving the reliability of the harvest as it was for boosting yield [25,26]. Consequently, crop water management could involve low-intensity watering practices that would not necessarily result in high levels of water availability. It is important therefore to detect different levels of water availability in order to identify low intensity watering practices. Such low level watering does not normally involve major irrigation infrastructure, but rather more ephemeral earthen channels, *shaduf*-type mechanisms, or simply watering ‘by hand’ using water-carriers, all of which are unlikely to leave recognisable material traces. Similarly, textual evidence although potentially a source of information regarding past water management is subject to a number of limitations, e.g. the inability to identify specific crops and watering practices, and biases, e.g. towards large-scale state controlled systems (for a discussion of these issues see [27,28]). Archaeobotanical remains are therefore a more promising source of evidence for these low level watering practices. For example, the weed seeds accompanying archaeological crops have been used as indicators of soil water availability [29–32]. An advantage of stable isotope analysis of the crop itself, however, is that water conditions can be inferred for specific crop remains, which cannot be achieved on the basis of textual evidence, architectural infrastructure or weed analysis (unless the weed seeds are associated with samples of a single crop). This permits the identification of differential treatment of crops, and potentially the importation of crops grown elsewhere, but cannot distinguish between water supplied through human agency and that derived from natural sources. This must be inferred from a comparison of crop water status with prevailing climate or from material evidence, such as the presence of irrigation structures.

In this paper we apply the results of this research to archaeobotanical crop remains. We have chosen sites from a range of climatic zones (spanning the Mediterranean to eastern Fertile Crescent, where water management (especially irrigation) was potentially practiced), and from a range of archaeological settings (encompassing small farming communities and major urban centres), and dating from the Neolithic to Islamic periods. This wide-ranging study of early agricultural practices, one of the largest of its kind, allows us to explore the varied factors that need to be taken into account when interpreting stable carbon isotope data. In total, 275 grain samples, from five crop genera and nine archaeological sites were analysed.

## Experimental Research on Present-Day Crops

In this article we refer to plant discrimination against ^13^C using the plant’s ∆¹³C value; which we define in section 3.2. The archaeobotanical stable carbon isotope data are interpreted on the basis of the crop water status ∆¹³C bands proposed by Wallace et al. [15]. The present-day studies underpinning this framework, and other comparable experiments [6–9], are primarily based on barley (*Hordeum* sp.) and free threshing wheats (bread wheat, *Triticum aestivum*, and durum wheat, *T*. *durum*). Several other crop species are, however, present in the archaeological material analysed here. Indeed most of the wheats at early agricultural sites are glume wheats (particularly einkorn, *Triticum monococcum*, or emmer, *T*. *dicoccum*).

The effect of crop water status on the ¹³C discrimination of glume wheats has, as yet, not been assessed but the ¹³C discrimination for the grain of glume wheats and free threshing wheats grown under the same water conditions have been reported. Khazaei et al. [33] reported that pot-grown einkorn had ∆¹³C values which differ by only 0.3‰ from those of durum wheat and by 0.6‰ from those of bread wheat. In a similar study, Konvalina et al. [34,35] reported similar (within 0.7‰) δ¹³C values for emmer and bread wheat. Larger discrepancies were reported by Heaton et al. [36] for einkorn grown at the Agios Mamas agricultural research station in Greece, which had ∆¹³C values differing by up to 2‰ from those of durum wheat grown under the same conditions. Heaton et al. [36] also reported differences in δ¹³C between the three glume wheat species (einkorn, emmer and spelt, *T*. *spelta*), grown at the John Innes Centre (Norwich, UK), although these differences tended to be smaller.

Stable carbon isotope experiments conducted on barley are complicated by the occurrence of two-row and six-row types (*Hordeum vulgare* var. *distichon* and *H*. *vulgare* var. *hexastichon* respectively). In present-day studies, two-row barley has ∆¹³C values about 1‰ higher than those of wheat grown under the same conditions [15,37–39]. Six-row barley produces even higher ∆¹³C values, which are about 1‰ higher than those of two-row barley [39,40] grown under the same conditions, and so 2‰ higher than wheat, as indicated by a comparison of research by Stokes et al. [13] and Flohr et al. [14]. The higher ∆¹³C values of six-row barley are assumed to be at least partly due to an increased use of carbohydrates formed early in the growth period, which are mobilised to grains to supplement grain filling [41–43].

Scant stable carbon isotope data are available for pulse crops (e.g. lentil, *Lens culinaris*; pea, *Pisum sativum*; and bitter vetch, *Vicia ervilia*). Most information has been derived from lentils grown experimentally alongside bread wheat in Syria [15]. These experiments indicated that the relationship between water availability and ∆¹³C in lentils is similar to that for wheat, although there was a broader range of ∆¹³C values for lentils than for wheat grown under a similar range of water conditions. Crop breeding studies, where bread wheat and lentils were grown together, also indicated that lentils had δ¹³C values within 0.2‰ of wheat grains [44,45]. The available information therefore suggests that lentils have similar ∆¹³C values to wheat, although the ∆¹³C of lentils seems to be lower than that of wheat when drought occurs late in the growth period [15].

## Materials and Methods

### Selection of sites and archaeobotanical samples

**Fig 1:**
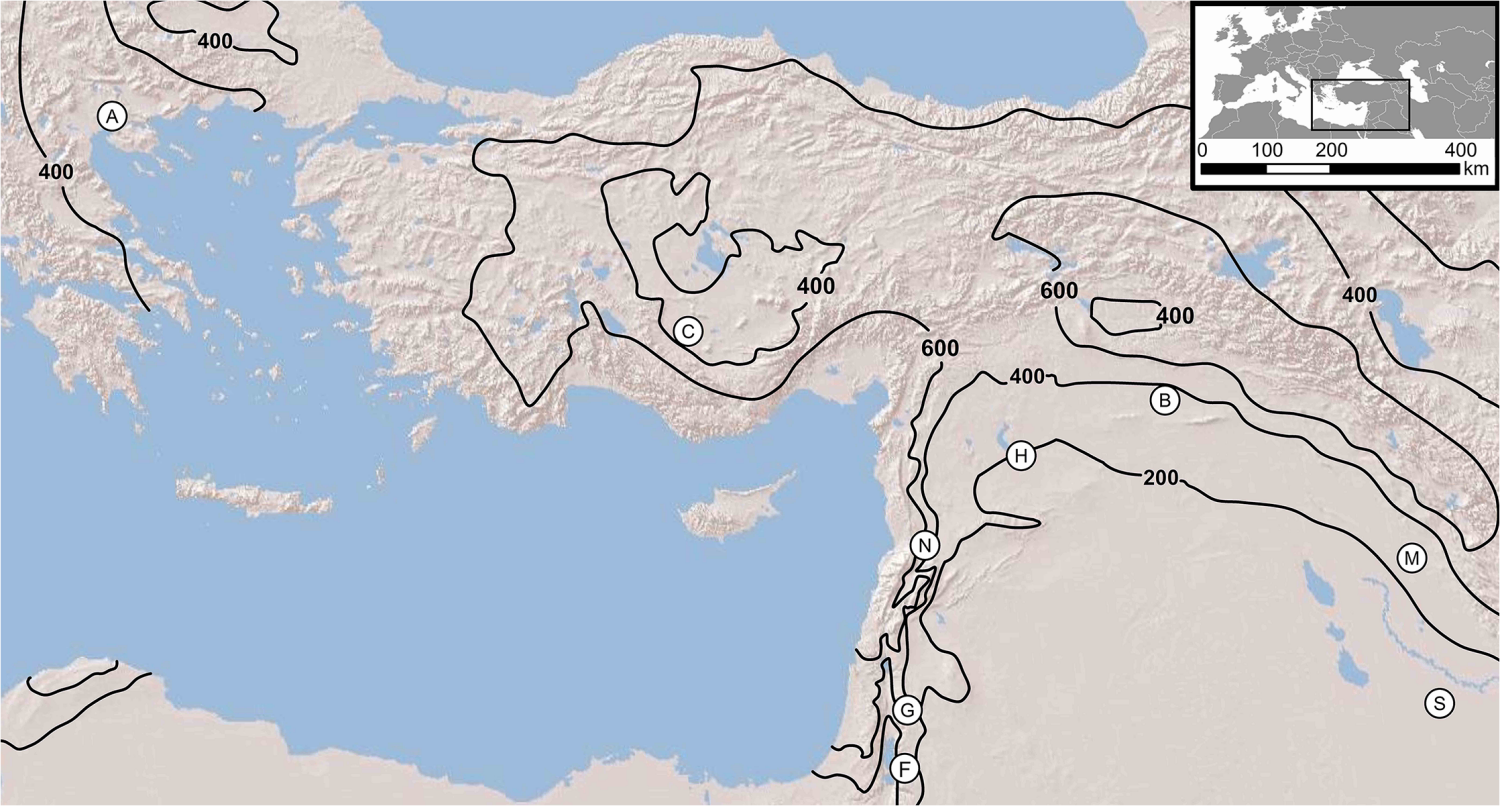
Map showing locations of archaeological sites with present-day major rainfall isohyets (mm per annum). Sites: A = Assiros Toumba, C = Çatalhöyük, F = Khirbet Fâris, G = Ain Ghazal, N = TellNebi Mend, H = Abu Hureyra, B = Tell Brak, M = Tell Madhur and S = Abu Salabikh. Shaded reliefbasemap used by permission. Copyright © 2009 Esri. All rights reserved. Rainfall data derived fromBioclim records for ~1950–2000 [79].

Archaeobotanical cereal grains and pulse seeds from nine archaeological sites were sampled for isotopic analysis. The sites date to the Neolithic or Bronze Age (Table 1), with the exception of Khirbet Fâris which dates to the Islamic period, and are located in southwest Asia (Fig 1), with the exception of Assiros Toumba in Greece. Key information and references for the sites studied are presented in Table 1. Full sample details are provided in the S1 Table, and accession of analysed material are held at the Institute of Archaeology, University of Oxford.

**Table 1:** Archaeological site information.

Site	Location	Occupation period (and period sampled, if different)	Predicted climatic conditions	Possible cultivation areas	Nearby rivers/water sources	Taxa analysed	References
Abu Hureyra	Central Syria	12000 – 5000 BCE	Arid to semi-arid	River valley and steppe	Euphrates + 2 minor tributaries	two-row barley	[80,81]
		(7000 – 5000 BCE)					
Ain Ghazal	Western Jordan	7000 – 6000 BCE	Semi-arid	River valley	Zarqa + Ain Ghazal spring	two-row barley; pea; bitter vetch	[67,82]
Çatalhöyük	Central Turkey	7400 – 5700 BCE	Semi-arid	Basin and hillsides	Flooding of Konya basin + Carsamba	einkorn wheat; emmer wheat; bread wheat; durum wheat; two/six-row barley; lentil; pea	[83–85]
		(6500 – 5700 BCE)					
Tell Madhur	Eastern Iraq	5300 – 4000 BCE	Arid to semi-arid	Basin	Diyala + 2 minor tributaries	six(?)-row barley	[46,86]
Tell Brak	North-east Syria	6600 – 900 BCE	Semi-arid	Wadi edges and steppe	Khabur + 2 minor tributaries	einkorn wheat; emmer wheat; two-row barley; lentil	[87–90]
		(4200 – 2300 BCE)					
Tell Nebi Mend	Western Syria	2700 – 1200 BCE,	Semi-arid	River floodplain	Orontes and Mukadiyah	emmer wheat; bread/durum wheat; two-row barley; lentil; bitter vetch	[91,92]
Abu Salabikh	Central Iraq	3000 – 2400 BCE	Arid	Irrigated plain	Irrigation canals from Euphrates	einkorn wheat; emmer wheat; two/six-row barley	[93–95]
Assiros Toumba	Northern Greece	1800 – 800 BCE	Sub-humid	Basin	Small stream	einkorn wheat; emmer wheat; spelt wheat, six-row barley; lentil; bitter vetch	[36,57,96]
		(c.1350 BCE)					
Khirbet Fâris	Western Jordan	400 – 1300 CE	Semi-arid	Wadi edges and plateau	Wadi Ibn Hammâd	bread wheat; durum wheat; two/six-row barley; lentil; bitter vetch	[56,97]

Samples from the earliest sites, Abu Hureyra and Ain Ghazal, date to the Pre-Pottery Neolithic B (PPNB), and derive from a range of primary (e.g. storage) and secondary (e.g. refuse) contexts. Çatalhöyük samples date to the late PPN (Pre-Pottery Neolithic) and early PN (Pottery Neolithic), and derive from concentrations of plant remains in buildings, e.g. on floor surfaces or in storage bins. The samples from Tell Madhur date to the 'Ubaid (Late Neolithic) period, and derive from a layer rich in barley grains on a floor, radiocarbon dated to 4470±80 cal. B.C. [46]. The samples from Tell Brak cover the entire Uruk (Chalcolithic to Early Bronze Age) period as well as the post-Uruk periods, hereafter referred to as Post-Uruk/Ninevite 5 and Post-Ninevite 5. Contexts sampled include both occupation deposits and pot contents. Samples from Abu Salabikh date to the late Uruk to Early Dynastic III periods, although the majority of samples are from the Early Dynastic III period, and a range of deposits was sampled including occupation debris, fire installations and concentrations of plant remains in an ash pit. Most of the samples from Tell Nebi Mend derive from pit fills and debris dated to the Bronze Age; the only exceptions are a few samples taken from the fill of an Iron Age oven. The samples from Assiros Toumba all derive from a single Late Bronze Age conflagration of a storeroom complex, dated to 1328±32 cal. B.C. [47]. The latest site, Khirbet Fâris, is an Islamic period site with samples dating to between the 5^th^ and 13^th^ Centuries A.D.; the majority of samples are from secondary contexts (such as fills and midden deposits) with a few samples from floor surfaces and oven fills.

Plant remains were retrieved by machine flotation at most sites (except for dense concentrations of grain found in storage contexts at Assiros Toumba, Tell Brak and Çatalhöyük which were processed by ‘hand’ flotation or dry sieving). Grain of cereal and/or pulse crops was identified, and the species analysed at each site are given in Table 1. At some sites, both glume wheats (einkorn, emmer or spelt) and free threshing wheats (bread wheat or durum wheat) were included in the analysis. For barley, it was not always possible to ascertain whether individual grains were of the two-row or six-row type, but the predominant type at each site is given in Table 1. Pulse seeds were available from fewer sites and included lentil, pea and bitter vetch.

### Carbon isotope analysis

Mass spectrometry to determine isotopic values was conducted at the NERC Isotope Geosciences Laboratory, Keyworth, and at the Oxford Radiocarbon Accelerator Unit. Experiments have shown that charring does not have a consistent or substantial effect on δ¹³C values [11,17,48], so no attempt was made to ‘correct’ δ¹³C values for charring. Contamination of archaeobotanical remains with non-structural carbon-containing compounds can produce spurious results. Acid-base-acid (ABA) chemical pre-treatment, routine in radiocarbon analysis [49], was utilised to remove carbonates and humic substances, the most likely sources of carbon-containing contaminants. Due to resource limitations, some samples were not ABA pre-treated, though at least a subset of samples was pre-treated for each site.

δ¹³C values were calculated to the Vienna Pee Dee Belemnite scale using within-run laboratory standard plant material calibrated against the universal standards NBS-19 and NBS-22. The δ¹³C value of atmospheric CO_2_, reconstructed from ice core bubbles, has decreased from c. -6‰, around 10,000 years ago [50,51], to c. -8‰ today [52]. The δ¹³C of atmospheric CO_2_ for the time periods covered by the archaeobotanical samples were therefore approximated by the AIRCO2_LOESS system [17]. ∆¹³C, the ^13^C discrimination independent of source CO_2_, was calculated following the equation developed by Farquhar et al. [9]:

$$\Delta^{13}\text{C}=\frac{\delta^{13}{\text{C}}_{air}-\delta^{13}{\text{C}}_{plant}}{1+\delta^{13}{\text{C}}_{plant}}.$$

## Results

### ABA pre-treatment

The ∆¹³C values of most samples tended to be slightly higher after pre-treatment (mean effect of pre-treatment = +0.18‰, n = 96), but this difference is not significant (p = >0.1). So ∆¹³C results from untreated material were used where no pre-treated samples exist. At Tell Nebi Mend, however, untreated and pre-treated ∆¹³C values from the same samples differed by a mean of 0.71‰ and so results for untreated sub-samples were excluded. This highlights the importance of routine pre-treatment for stable carbon isotope analysis, as recommended by Fraser et al. [48].

### Wheat (Table 2 and Fig 2)

At the Assiros Toumba, Tell Brak and Abu Salabikh, the mean ∆¹³C value for (glume) wheat is indicative of well-watered crops. These three sites are located in contrasting climatic zones. Assiros Toumba is located in the wettest climate, and so the high ∆¹³C values could simply be a product of dry farming in a moist location. Abu Salabikh is located in an area where today’s climate is arid and, although slightly wetter in the past [53,54], dry farming is unlikely to have been feasible there during the Bronze Age [55]. Thus, the high level of water availability reflected in the high ∆¹³C values at Abu Salabikh indicates deliberate irrigation. Tell Brak is located in a less arid climatic zone than Abu Salabikh, but the site would still have been marginal for dry farming, despite conditions having been slightly wetter during the site's occupation than today [46,47]. ∆¹³C values indicative of well-watered crops at this site could therefore be explained by cultivation of naturally moist soils near rivers, watering ‘by hand’, or flood irrigation.

**Fig 2:**
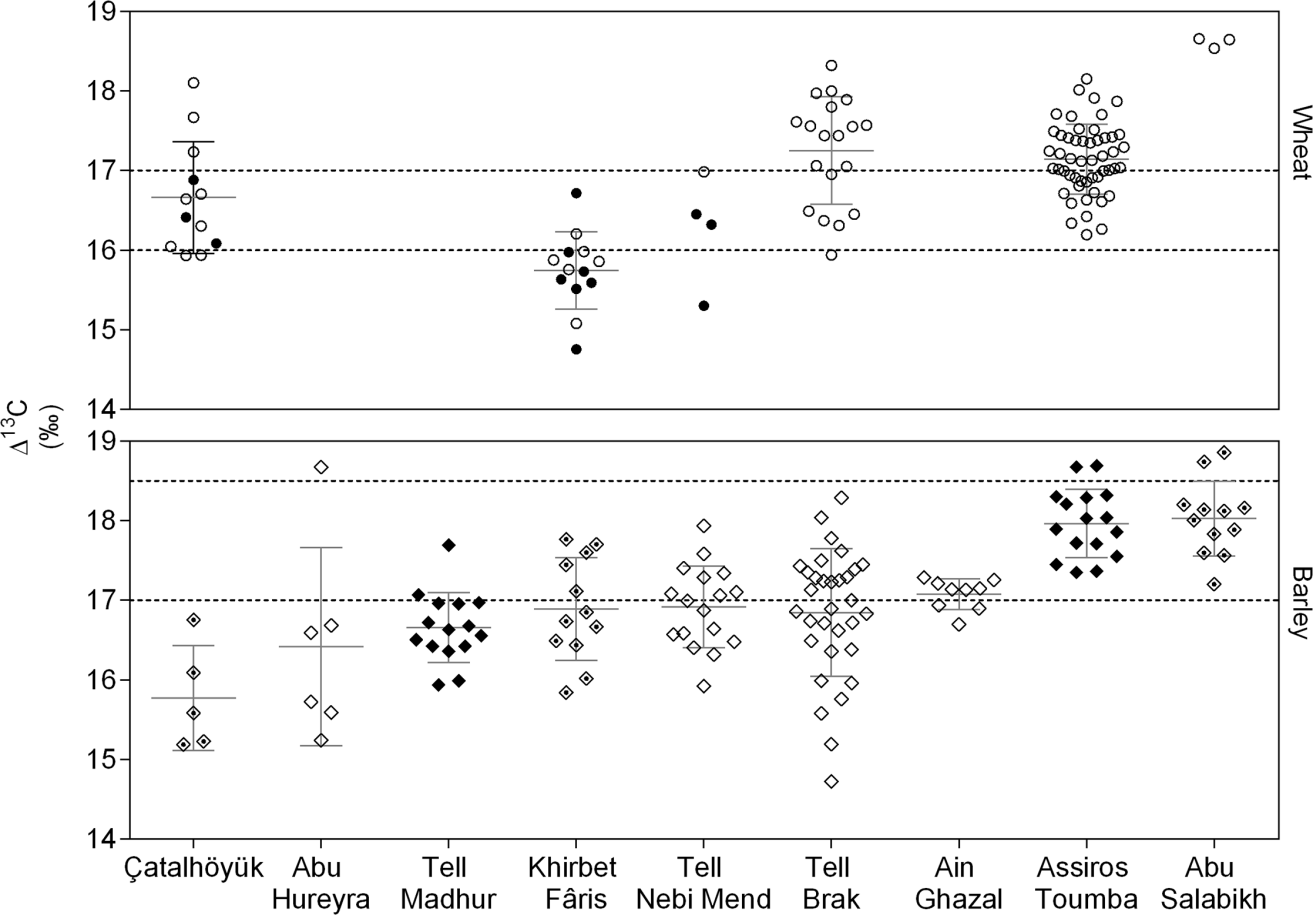
Δ¹³C results for cereal grains. Dashed lines indicate the suggested 'boundaries' betweenΔ¹³C ranges indicative of crops grown under poorly (low Δ¹³C), moderately, and well (high Δ¹³C) watered conditions, based on the analysis of present-day crops [10]. For wheat, this is based on free-threshing wheat, for barley on the mean values for two‐ and six‐row barley. Bars indicate means and standard deviations. **○** = glume wheat, ● = free threshing wheat, **◇** = two-row barley, ◆ = six-row barley, and ◈ = two/six-row barley.

**Table 2:** ∆¹³C results.

Site / Taxon	Number of samples	Mean ∆¹³C (‰)	Standard deviation (‰)	Min. (‰)	Max. (‰)
**Abu Hureyra**					
Two-row barley	6	16.42	1.24	15.24	18.68
**Ain Ghazal**					
Two-row barley	9	17.08	0.19	16.70	17.29
Lentil	2	-	-	15.71	15.96
Pea	5	16.07	0.69	15.35	17.11
**Çatalhöyük**					
Glume wheat	9	16.73	0.79	15.93	18.10
Free threshing wheat	3	-	-	16.09	16.88
Two/six-row barley	5	15.77	0.66	15.19	16.75
Lentil	2	-	-	16.80	17.28
Pea	6	15.85	0.92	14.55	17.25
**Tell Madhur**					
Six(?)-row barley	15	16.66	0.44	15.93	17.69
**Tell Brak**					
Glume wheat	19	17.25	0.67	15.94	18.32
Two-row barley	32	16.85	0.80	14.72	18.29
Lentil	7	17.41	0.76	16.36	18.42
**Tell Nebi Mend**					
Glume wheat	1	16.98	-	-	-
Free threshing wheat	3	-	-	15.30	16.45
Two-row barley	17	16.92	0.51	15.92	17.94
Lentil	4	-	-	16.83	18.77
Pea	1	16.31	-	-	-
Bitter vetch	2	-	-	17.12	18.77
**Abu Salabikh**					
Glume wheat	3	-	-	18.54	18.66
Two/six-row barley	12	18.03	0.47	17.20	18.86
**Assiros Toumba**					
Glume wheat	51	17.14	0.44	16.19	18.15
Six-row barley	16	17.97	0.43	17.35	18.69
Lentil	2	-	-	16.50	19.86
Bitter vetch	3	-	-	18.14	19.90
**Khirbet Fâris**					
Free threshing wheat	13	15.74	0.48	14.75	16.72
Two/six-row barley	12	16.89	0.65	15.84	17.77
Lentil	3	-	-	15.24	15.52
Bitter vetch	6	15.92	0.40	15.36	16.53

The ∆¹³C values for wheat grain (105 samples from 8 sites) are mostly between 16.2‰ and 17.7‰ (mean ±1σ). In terms of the water status framework based on stable isotope analysis of present-day crops [15], this range encompasses moderately watered crops (>c.16‰) and well-watered crops (>c.17‰).

**Fig 3:**
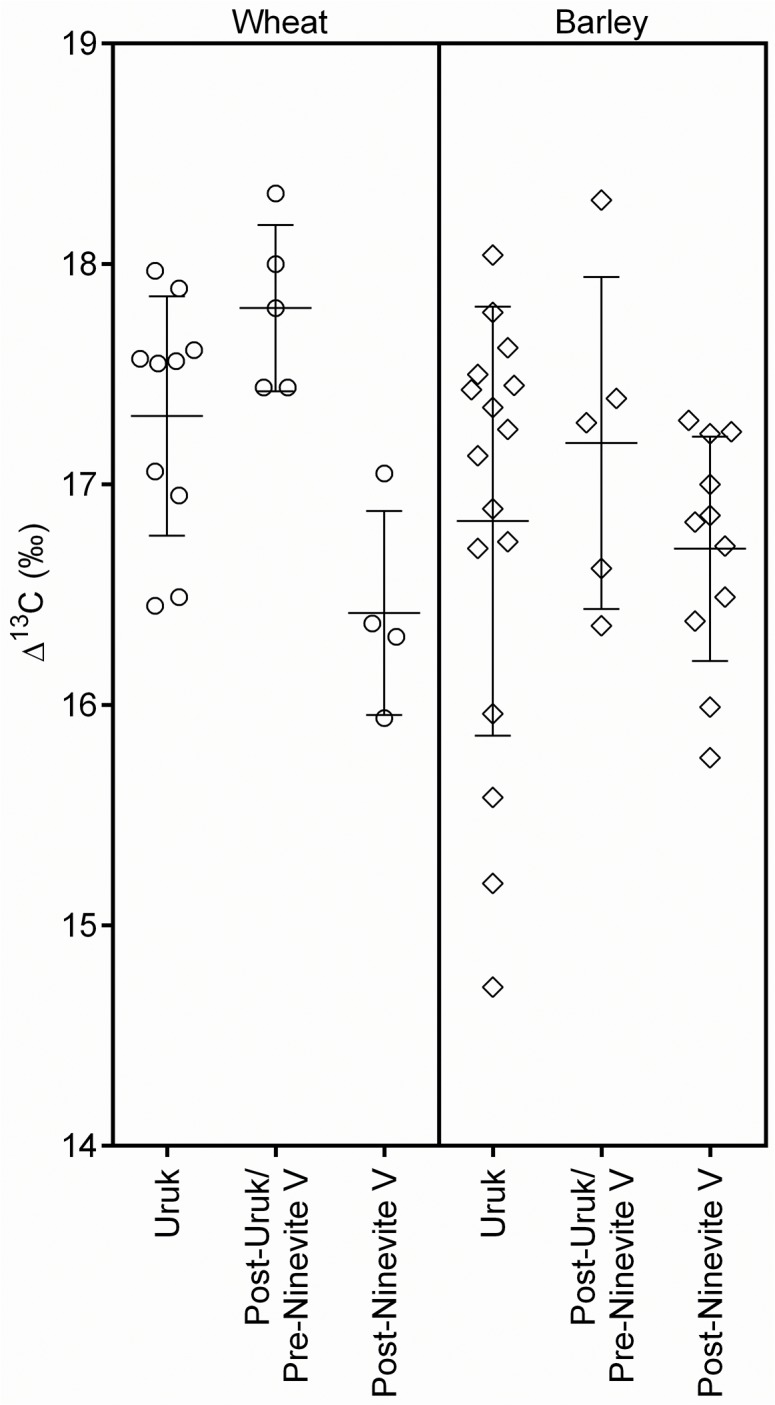
∆¹³C results for cereals grains from Tell Brak samples grouped by chronological period. Bars indicate means and standard deviations. **○** = glume wheat and **◇** = barley.

Variation in the glume wheat ∆¹³C values in the Assiros Toumba storerooms is consistent with that expected for multiple crop growing locations in a single year [36]. The large variation in ∆¹³C for the Tell Brak glume wheat is unsurprising given the site's large hinterland and long occupation. The mean ∆¹³C for the Post-Uruk/Ninevite 5 samples at Tell Brak is slightly higher than that for the Uruk samples (difference = 0.50‰, p = 0.065) and substantially higher than that for the Post-Ninevite 5 period (difference = 1.38‰, p = 0.003) (Fig 3). Only three Abu Salabikh (glume) wheat samples were analysed, and all produced similar results.

The wheat from two of the remaining sites, Çatalhöyük and Tell Nebi Mend, has a mean ∆¹³C value between 16‰ and 17‰, indicating a moderate water status. The mean ∆¹³C values for free threshing wheat and glume wheat from Çatalhöyük were similar but the variation between samples of glume wheat is high, suggesting that this type of wheat, at least, may have been grown under varied water conditions. There is a large variation between the ∆^13^C values of the four samples from Tell Nebi Mend, with the Early Bronze Age sample (free threshing wheat) having the lowest value and the latest Iron Age sample (glume wheat) the highest.

Only one site, Khirbet Fâris, had wheat grains, both bread wheat and durum wheat, with a mean ∆¹³C value in the poorly watered range (<16‰). As this is the youngest of the sites, climate aridification, which has continued since the early Holocene climatic optimum, may explain the lower ∆¹³C values. On the basis of the weed assemblage at Khirbet Fâris, Hoppé [56] suggested that some crops may have been irrigated in the 13^th^ Century AD. The samples analysed here indicate, at most, moderate levels of irrigation for the same period.

### Barley (Table 2 and Fig 2)

The ∆¹³C values for barley (127 samples from 9 sites) are mostly between 16.2‰ and 17.9‰ (mean ±1σ), which is a very similar range to that for wheat. As barley is known to produce ∆¹³C values 1–2‰ higher than wheat when grown under the same conditions (see above), their similar ∆¹³C ranges indicate that the barley was mostly grown under drier conditions than wheat.

At none of the sites, does the mean ∆¹³C value of barley suggest that it was well-watered (taking the threshold for well-watered barley to be a mean (18.5‰) of that for two- and six-row barley). At Assiros Toumba and Abu Salabikh, however, the ∆¹³C values are close to the minimum expected for well-watered two-row barley (18‰). The wheat at both sites was also found to be well-watered. At Assiros, the mean ∆¹³C for barley is 0.83‰ higher than that of wheat, which is similar to the difference reported between present-day wheat and two-row barley. The barley from Assiros Toumba, however, is of the six-row type [57], for which even higher ∆¹³C values would be expected if the wheat and barley were grown under the same conditions. It is possible, therefore, that barley was grown under drier conditions than wheat at Assiros Toumba. At Abu Salabikh, the ∆¹³C values for barley are sufficiently high to imply irrigation, given the site’s arid setting. The mean ∆¹³C of barley, however, is lower than that of wheat, and so barley was apparently less intensively irrigated than wheat.

The barley from Tell Brak has a moderate-poor water signal. Again, barley has lower ∆¹³C values than wheat, which strongly indicates that barley was less well-watered than wheat. Barley ∆¹³C values are highly varied, as they are for wheat. The difference in mean barley ∆¹³C values between the different chronological periods is smaller and less significant than that for wheat (Uruk to Post-Uruk/Ninevite 5 difference = 0.35‰, p = 0.42; Post-Uruk/Ninevite 5 to Post-Ninevite 5 difference = 0.48‰, p = 0.25) (Fig 3). The remaining sites also have barley ∆¹³C values in the moderate-poor water status range. At Khirbet Fâris, the mean ∆¹³C of barley is 1.15‰ higher than that of wheat, indicating that these two cereals were grown under similar water conditions. Barley ∆¹³C values for Çatalhöyük indicate somewhat drier conditions than at the other sites and, on average, 0.89‰ lower than those for wheat at the site. Barley samples from Abu Hureyra exhibit greater variation in ∆¹³C, implying a diversity of growing conditions, while there is very little variation in the ∆¹³C values of the barley samples from Ain Ghazal.

### Pulses (Table 2 and Fig 4)

The ∆¹³C values for lentils (20 samples from 6 sites) are between 15.8‰ and 18.3‰; for peas (7 samples from 2 sites) between 15.2‰ and 16.7‰; and for bitter vetch (16 samples from 4 sites) between 15.5‰ and 18.8‰. Pulses with ∆¹³C values predominantly in the range indicative of well-watered crops (based on the analysis of modern-day lentils) were found at three sites: Assiros Toumba, Tell Brak and Tell Nebi Mend. Assiros Toumba and Tell Brak also produced well-watered cereals. The ∆¹³C values for lentil and bitter vetch at Assiros Toumba are consistently high, with just one lentil sample in the moderately watered range, and also slightly higher than those for wheat at the site, possibly indicating supplementary watering of pulses. The ∆¹³C values for lentils at Tell Brak are slightly lower and less varied than those for the Assiros Toumba pulses, but still primarily in the well-watered range. These values suggest that the Tell Brak pulses received similar amounts of water to the wheat at the site. The pulses (lentil, bitter vetch and pea) from Tell Nebi Mend also have ∆¹³C values similar to those of wheat from the site.

**Fig 4:**
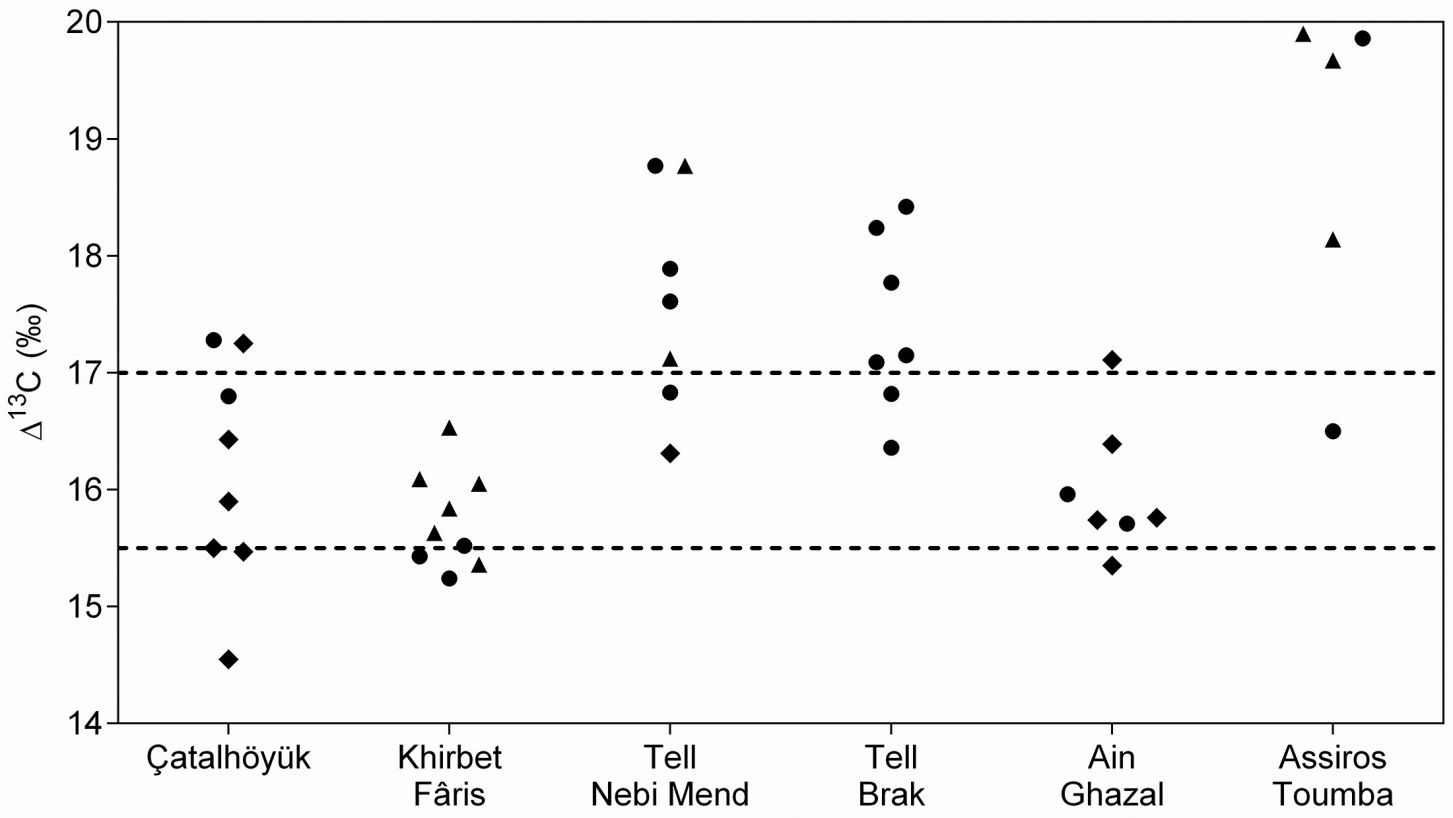
∆¹³C results for pulse seeds. Dashed lines indicate the suggested 'boundaries' between ∆¹³C ranges indicative of lentils grown under poorly (low ∆¹³C), moderately, and well (high ∆¹³C) watered conditions, based on the analysis of present-day crops [15]. ● = lentil (Lens culinaris), ◆ = pea (Pisum sativum), ▲ = bitter vetch (Vicia ervilia).

Pulse samples from the other three sites produced ∆¹³C values that are mostly indicative of drier conditions. The ∆¹³C values for lentil and bitter vetch at Khirbet Fâris are low, and very similar to those for the wheat at the site, suggesting that both cereals and pulses were poorly watered. The lentil and pea at Ain Ghazal have ∆¹³C values very similar to the Khirbet Fâris pulses, except for one lentil sample which has a somewhat higher value. The ∆¹³C values for pea at Çatalhöyük are more varied, perhaps indicating a diversity of growing conditions for this pulse at least, extending to conditions drier than those in which the wheat was grown.

## Discussion

Crop ∆¹³C reflects the plant's water status during its life-cycle, which is influenced by both natural water sources (e.g. precipitation) and water added through human agency (e.g. irrigation). It can therefore be used to provide information on crop growing conditions in the past, including choice of soils for cultivation and water management practices, provided it is interpreted in the context of local environmental conditions and past climate. There is palaeoclimatic evidence to suggest that the eastern Mediterranean was naturally moister in the early Holocene than the present [53,54], and there may have been precipitation in the summer months [58,59]. Our approach therefore attempts to disentangle natural and anthropogenic water sources by considering ∆¹³C results in their local context, and taking past climate into account by making inter-species comparisons between contemporary crops.

### Irrigation of cereals?

Well-watered wheat has been identified at Abu Salabikh, Assiros Toumba and Tell Brak. The highest ∆¹³C values for wheat (indicating the wettest growing conditions) were for crops from Abu Salabikh (Iraq), which is situated in the driest climatic zone of the sites studied, so climate cannot explain these results. The ∆¹³C values for wheat from Assiros Toumba and Tell Brak are very similar but Assiros is located in a sub-humid zone and Brak in a semi-arid zone.

Abu Salabikh is located along one of the branching irrigation canals ultimately derived from the Euphrates, and crossing Mesopotamia [60], in a highly arid region. Thus, the most likely explanation for the very high ∆¹³C values for wheat grain at the site is that the wheat was irrigated. It is more difficult to say whether any water management was practised at Assiros Toumba or Tell Brak. At Assiros Toumba naturally moist soils could have been found in the basin in which the site is located, and there is no structural evidence of irrigation. On the other hand, small-scale irrigation or hand watering could have been applied to wheat grown on drier soils without leaving any material trace. At Tell Brak the need for irrigation and its feasibility have been debated [61,62] with no firm conclusions. Naturally moist soils are located in the hinterland of Tell Brak along the wadis to the east and south of the site [63], and the River Jaghjagh or nearby wadis could also have been used as a source of irrigation waters [64]. At the remaining sites, only the wheat at Çatalhöyük exhibits ∆¹³C values primarily in the moderate to well-watered range, indicating that at none of these sites is there evidence for irrigation.

### Cereal cultivation and land-use

The samples from Assiros are unusual in that they derive from a single conflagration of a storeroom complex, and the ∆¹³C variation of the wheat is consistent with a single year’s harvest from several locations [36]. The barley from the site displays a similar degree of variation which is again in keeping with this. The greatest variability in ∆¹³C values is seen in the barley from Tell Brak and Abu Hureyra, both of which were occupied for a long period, with access to a variety of environments [63,65]. At Tell Brak this is unlikely to be explained by long-term climatic change because the variation in barley ∆¹³C values within all three periods is high, and the difference between periods not statistically significant. The ∆¹³C values for wheat in the Post-Uruk/Ninevite 5 period, however, are higher and less varied than in the Uruk period, the ∆¹³C values for wheat in the Post-Ninevite 5 period are significantly lower than in the Post-Uruk/Ninevite 5 period. This suggests chronological changes unrelated to climate, as climatic change would be expected to affect both crops. The consistently well-watered ∆¹³C signal for wheat in the Post-Uruk/Ninevite 5 period may therefore indicate a change in agricultural practice, which agrees with weed evidence that indicates that the site’s glume wheat were grown in better-watered fields in the Post-Uruk/Ninevite 5 period [64]. If so, these practices seem to have been short-lived because there is an apparent decline in water availability in the Post-Ninevite 5 period (a period for which no weed evidence is available). Variation in barley ∆¹³C values is more likely explained by year-to-year variations in weather conditions and/or watering practices, or the pooling of crops from a large catchment area, incorporating varied microclimates, levels of natural moisture, and/or watering strategies. The smaller number of samples from Abu Hureyra do not permit a comparison between chronological periods so, while the high ∆¹³C variation at this site may be explained by the cultivation of the naturally moist soils of the floodplain as well as drier soils fed by small streams on the steppe, long- or short-term temporal changes in natural conditions or watering practices are also possible.

At the opposite end of the spectrum, the barley from Neolithic Ain Ghazal exhibits a very tight range of ∆¹³C values, with variation as low as that found for modern barley that was all grown under the same water conditions (e.g. [15]). The samples were not deposited in a single event so it seems that farmers had significant control over water availability, possibly mediated through use of the spring and floodplains in the immediate vicinity of the site [66–68].

At the remaining sites (Tell Madhur, Tell Nebi Mend and Khirbet Fâris), the variation in ∆¹³C values is less extreme and the values tend to be in the poor to moderately watered range. Tell Madhur is located some distance from the nearest rivers and wadis that could have provided naturally moist soils or an opportunity for irrigation, but Tell Nebi Mend’s location at the confluence of two rivers provides access to a fertile floodplain. So, while the low ∆¹³C values are consistent with cultivation of soils in the immediate vicinity of Tell Madhur, the low values at Tell Nebi Mend suggest that the soils adjacent to the site were not used for cereal (barley) cultivation. The low ∆¹³C values for Khirbet Fâris are consistent with crops receiving low-levels of irrigation in the steep-sided wadi to the west of the site.

### Differential treatment of crops

**Fig 5:**
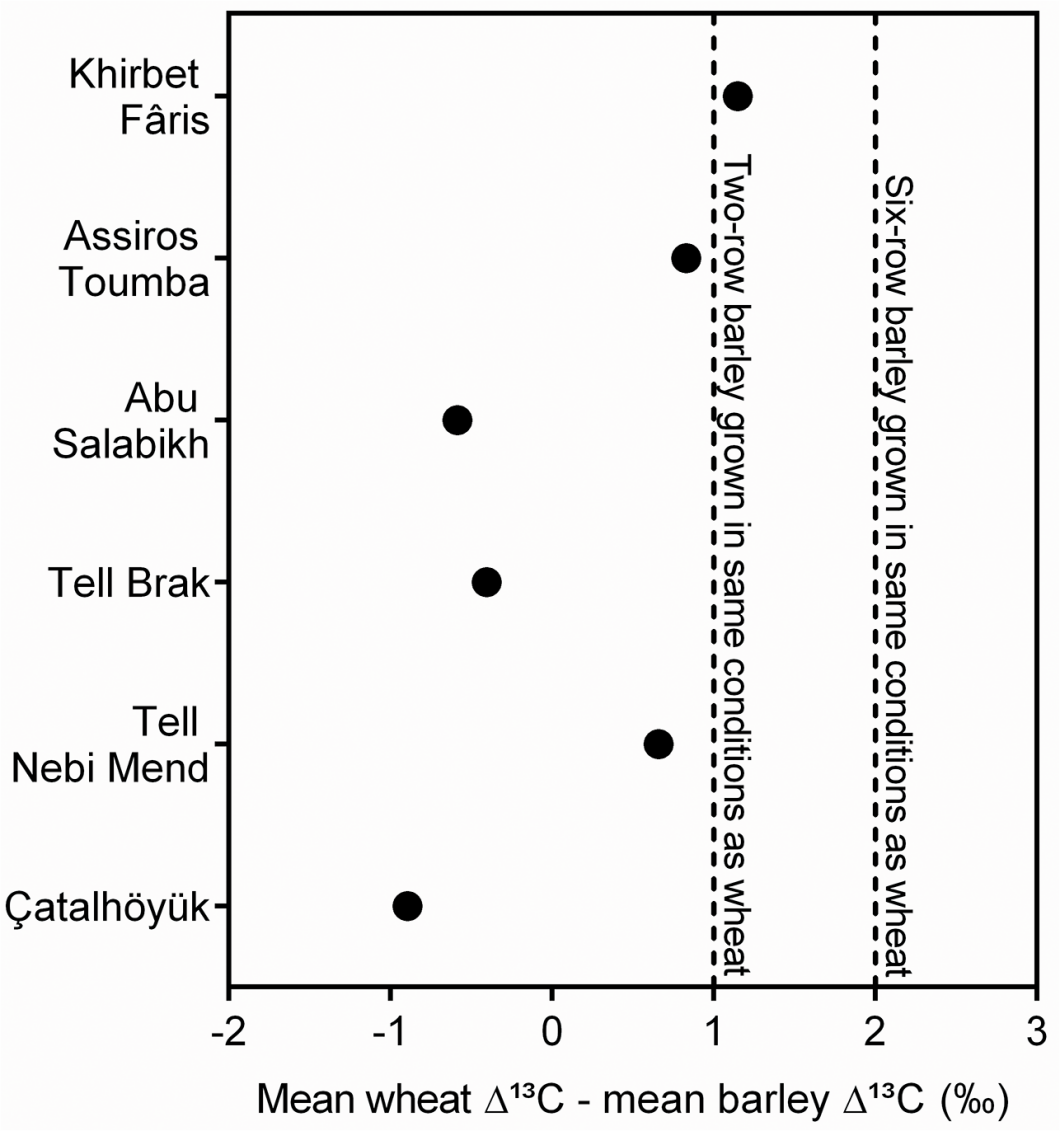
Difference between mean ∆¹³C for barley grain and mean ∆¹³C for wheat grain at each site. Dashed lines indicate the ∆¹³C difference predicted if two-row barley (at +1‰) or six-row barley (at +2‰) were grown with the same water availability as wheat, based on the analysis of present-day crops [15].

Of the six sites for which both wheat and barley samples were analysed, three (Tell Brak, Çatalhöyük and Abu Salabikh) have ∆¹³C values for barley that are mostly lower than those for wheat (Fig 5), whereas higher values for barley would be expected if both crops were grown under the same conditions. This provides a strong indication that barley tended to be grown under drier conditions than wheat. Even at the other three sites (Assiros Toumba, Tell Nebi Mend and Khirbet Fâris), barley ∆¹³C values are not sufficiently greater than those of wheat to suggest that barley was grown under wetter conditions. So it seems that barley was usually grown under drier, or sometimes similar, water conditions to wheat. While it is conceivable that this difference between wheat and barley ∆¹³C values is due to greater water use efficiency in modern barley (but not modern wheat), barley samples with ∆¹³C values in excess of 20% are reported for Neolithic sites in central Europe [69], where water availability is known to have been high, suggesting that our results reflect genuinely drier growing conditions for barley than wheat.

The comparison of wheat and barley ∆¹³C values from Khirbet Fâris suggests that they were grown under very similar conditions, which is consistent with rain-fed or poorly irrigated crops. At Assiros Toumba, where the ∆¹³C results are also consistent with rain-fed cultivation, the difference in ∆¹³C between wheat and barley is of the order of 1‰, whereas a difference of 2‰ might be expected for the six-row barley grown at Assiros Toumba, which may suggest low-level watering or the choice of moister soils for the cultivation of wheat.

At Abu Salabikh, on the other hand, the evidence that wheat was grown under wetter conditions than barley is particularly strong. As cereal irrigation has been established here [70], the most likely explanation is that greater levels of irrigation were applied to the wheat than the barley. The difference in ∆¹³C values could be a direct effect of the quantity of water or number of irrigations applied to each crop or it may reflect the cultivation of barley on land suffering from high salinity due to irrigation, which reduces water availability thus lowering ∆¹³C.

At Tell Brak and Tell Nebi Mend, where there is little or no archaeological evidence for cereal irrigation, the difference in ∆¹³C between wheat and barley may be explained by the growing of barley in areas that were naturally drier than those used for wheat production, or by the small-scale addition of water to the areas of wheat cultivation. As both sites are located in a semi-arid environment, the higher ∆¹³C values for wheat at Tell Brak are perhaps more likely to be the result of added water, especially in the post-Uruk period. The discrepancy in ∆¹³C values between wheat and barley is greatest at Çatalhöyük. This may suggest that the wheat was irrigated and the barley not, or that both crops were unirrigated but the wheat grown under naturally moister conditions within the mosaic landscape of the Konya plain, where both moist and dry land would have been within easy reach of the site [71,72].

The pulse crops at some sites exhibit large variation in ∆¹³C values which cannot be attributed to species differences. The consistently low values of pulse ∆¹³C at Khirbet Fâris and Ain Ghazal indicates poor to moderate water availability, while at Çatalhöyük, where the ∆¹³C values are more variable, greater levels of watering of some of pulse crops may be indicated. At Tell Brak and Tell Nebi Mend, where the both the mean ∆¹³C and the variability are high, watering of pulses in these arid environments is likely but to varying degrees. Even at Assiros Toumba, in a relatively moist area, watering of some pulse crops may be indicated by the extremely high ∆¹³C values of some samples. The varying levels of water availability at some sites may be due to a number of factors, such as changing agricultural practices (e.g. at Tell Brak) or the cultivation of pulses in gardens subject to variable practices (e.g. at Assiros Toumba).

## Conclusion

The results presented here represent one of the largest collections of ∆¹³C values for archaeological crop remains both in terms of numbers of sites and numbers of samples. The interpretation of these results has drawn on both the archaeological and environmental context of the sampled crops to distinguish between the effects of natural conditions (both local and regional) and human-mediated interventions (such as choice of soils and irrigation or watering). This has been facilitated by the direct assessment of ancient crop water status on an absolute scale of water availability based on present-day reference material [15].

The most striking overall trend in the results is the tendency for wheat to have been grown under wetter conditions than barley in western Asia. This differential treatment implies that greater measures were taken to ensure a successful wheat harvest, potentially at the expense of barley. For pulse crops our data are more limited, but there is evidence to suggest that pulses were grown in more variable conditions than the cereals, which may indicate opportunistic watering. Our ability to identify trends such as these enables us to address archaeological questions regarding the production and consumption of crops in both the prehistoric and historic periods. In the latter case the wealth of documentary evidence regarding the selection of crops [73–78] can be tested against evidence from the direct analysis of crop remains. This would require a detailed contextual comparison of the archaeobotanical and textual evidence which, while beyond the scope of this paper, has the potential to significantly advance our knowledge of agricultural economy in proto- and early urban societies.

## Supporting Information

S1 Table δ¹³C result, ∆¹³C calculation, chemical pre-treatment status and archaeological accession information for all analysed samples. (CSV)
